# Significant Hall–Petch effect in micro-nanocrystalline electroplated copper controlled by SPS concentration

**DOI:** 10.1038/s41598-023-27669-2

**Published:** 2023-01-09

**Authors:** Yu-Jyun Kao, Yu-Ju Li, Yu-An Shen, Chih-Ming Chen

**Affiliations:** 1 Department of Chemical Engineering, National Chung Hsing University, 145 Xingda Rd., South Dist., Taichung, 402 Taiwan; 2 Department of Materials Science and Engineering, Feng Chia University, No. 100, Wenhwa Rd., Seatwen, Taichung, 407 Taiwan; 3 Innovation and Development Center of Sustainable Agriculture (IDCSA), National Chung Hsing University, 145 Xingda Rd., South Dist., Taichung, 402 Taiwan

**Keywords:** Electronic materials, Mechanical properties, Mechanical properties, Metals and alloys

## Abstract

Electroplated Cu has been extensively applied in advanced electronic packaging, and its mechanical properties are critical for reliability. In this study, Cu foils fabricated through electroplating with various bis-(3-sulfopropyl) disulfide (SPS) concentrations are examined using tensile tests. The SPS concentration affects the grain size of the electroplated Cu foils, resulting in different mechanical properties. A significant Hall–Petch effect, ${\sigma }_{y} = 197.4 + 0.12{d}^{\frac{-1}{2}}$, is demonstrated for the electroplated Cu foils. The different concentrations of impurities identified through time-of-flight secondary ion mass spectrometry correspond to the different grain sizes, determining the transgranular and intergranular fracture during the tensile test. The results demonstrate that the SPS concentration controlling the microstructures of the electroplated Cu results in a Hall–Petch effect on the mechanical properties of the electroplated Cu foils.

## Introduction

In the past, aluminum was used as the primary interconnecting material in electronic packaging; however, the high demand for interconnecting materials with the development of advanced electronic packaging has led to the replacement of aluminum with copper (Cu). This is because Cu exhibits better electrical conductivity and resistance to electromigration than aluminum. Additionally, the excellent thermal conductivity, ductility, relatively high melting temperature, and appropriate strength of Cu have made it a popular conductor material in electronic products^1,2^.

Electroplating of Cu is important for industrial mass production in the fabrication of conductive traces, wires, and metallization in electronic devices^3–5^. Currently, most electroplating solutions for semiconductor and printed-circuit board factories are commonly composed of sulfuric acid and copper sulfate because of their low toxicity and excellent management of the plating baths^5–7^. In contrast, organic additives added to the electroplating solutions are vital in controlling the deposition rate of reduced Cu atoms and microstructures of the electroplated Cu. For instance, some additives in the plating solutions can be used to fabricate Cu films with nanotwin structures to enhance their electricity, strength, and void suppression^5,8,9^. One of the additives is the chloride ion (Cl^−^) from NaCl or HCl, which increases the reduction rate of Cu ions^10^. In addition, Cl^−^ can co-work with other additives, such as polyethylene glycol (PEG), to suppress the rate of Cu reduction on the cathode surface^11,12^. Bis-(3-sulfopropyl) disulfide (SPS) reacts with Cl^−^ to accelerate the reduction rate of Cu ions on the cathode surface and reduce the surface roughness of the electroplated Cu^13^. The variation in the concentrations of the additives significantly affected the microstructures of the electroplated Cu because of the change in the deposition kinetics of the reduced Cu atoms^14^. Therefore, the influence of the concentration of the additives on the properties of electroplated Cu is worth investigating.

In recent years, three-dimensional integrated circuits have become an essential solution for fabricating high-performance electronic products with extreme miniaturization^15,16^. Electroplated Cu has been widely applied in redistribution layers (RDLs) and through-silicon vias (TSVs) in advanced electronic packaging such as fan-out wafer-level packaging^17,18^. In RDLs and TSVs, the Cu wires must pass through silicon wafers and polymer substrates (epoxy molding compound). The latter exhibits a high thermal expansion, whereas the thermal expansion of the former is very low, and that of Cu ranges between them. Thermal stress is generated in the Cu wires by the different coefficients of the silicon, Cu, and epoxy molding compound during the thermal cycling tests^19,20^. Recently, the size of Cu wires in semiconductor chips has been reduced to the nanoscale, and their excellent mechanical properties have become increasingly important^21–23^.

As mentioned earlier, the strength of Cu is critical in advanced electronic packaging, and the formulation is the key in changing the microstructure of the electroplated Cu, which is significantly related to the mechanical properties. However, the effect of the SPS concentration on the mechanical properties of Cu has not been extensively studied. In this study, we fabricated electroplated Cu films using electroplating solutions with specific concentrations of PEG and Cl^−^ and different concentrations of SPS, and their mechanical properties were evaluated using tensile tests.

## Experimental procedures

### Electroplating Cu film

**Figure 1:**
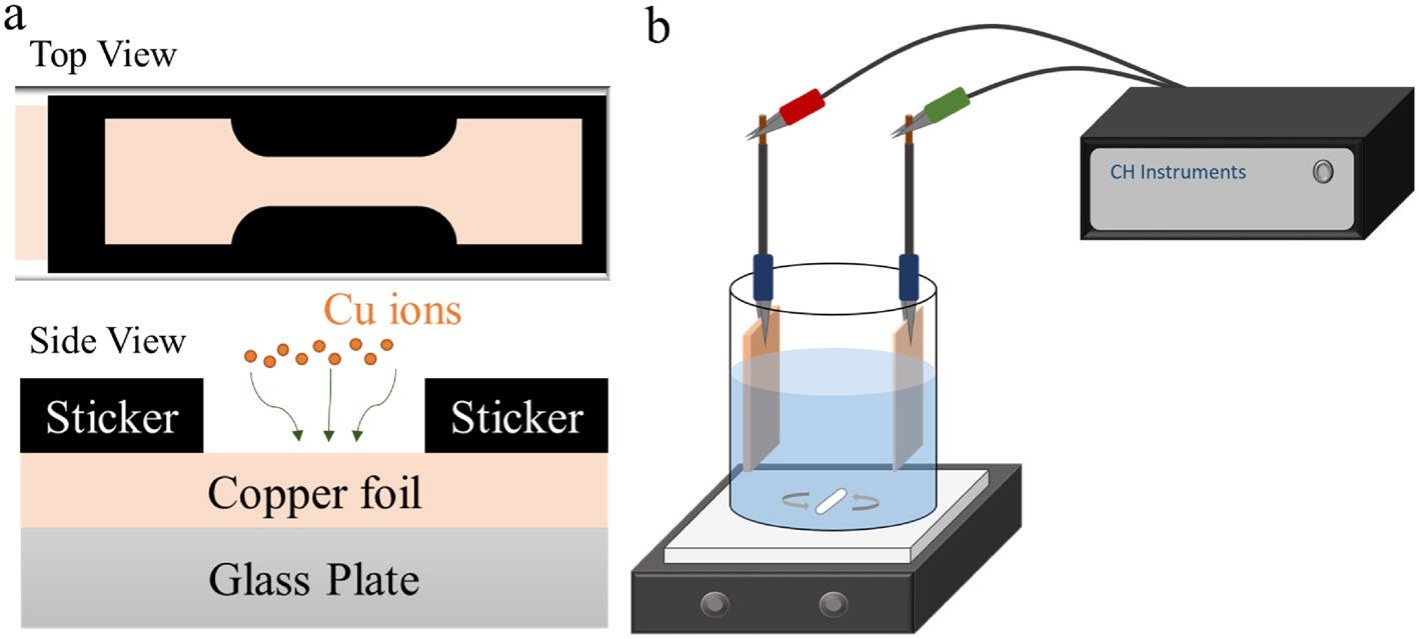
(**a**) Top-view and side-view structures of the electroplated substrate at the cathode and (**b**) a schematic of the electroplating facility.

A glass plate affixing a Cu foil (Alfa Aesar, 99.8% purity, 25 μm thick) and an acid-resistant tape with a dog-bone shaped space area was used as the substrate for the electroplating of Cu at the cathode of the electroplating bath (Fig. 1a). The anode of the electroplating bath was a Cu-0.04 wt% P plate cleaned using sulfuric acid (2 vol%) and dilute hydrogen peroxide. The electrolyte mainly consisted of high-purity CuSO_4_·5H_2_O and 5 vol. % H_2_SO_4_ (purity: 95–98%). The electroplating solutions comprised the electrolyte, 60 ppm Cl^−^, 50 ppm PEG, and 0–2.0 ppm SPS for fabricating the electroplated dog-bone shaped Cu films. A potentiostat (CHI-611E, CH Instruments, Austin, USA) controlled the direct current under a current density of 4 ASD, and a magnetic stirrer provided mechanical stirring at 1000 rpm to fabricate a uniform electroplated Cu, as shown in Fig. 1b. According to the electroplating rates, the thickness of the Cu films was set at approximately 50 μm.

### Tensile test

**Figure 2:**
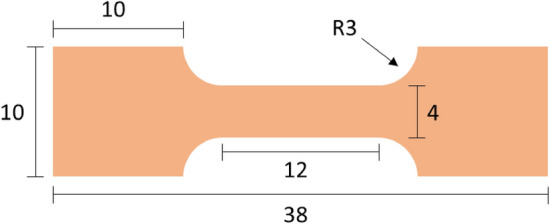
Dimension of the dog-bone shaped electroplated Cu for the tensile tests (unit: mm).

After electroplating, the electroplated Cu samples were carefully removed from the substrates. Figure 2 shows the dimensions of the electroplated Cu samples used in the tensile tests. A universal tester (AGS-X, SHIMADZU, Kyoto, Japan) was used to conduct tensile tests at a strain rate of 0.6 mm/min. The autographed stress–strain curve of each test demonstrated the ductility and yield strength of each electroplated Cu sample. A scanning electron microscope (SEM, JEOL JSM-7800F, Japan) was used to capture the top-view morphologies of the electroplated Cu foils before and after the tensile test. An electron backscatter diffraction (EBSD, Oxford, UK) set on the SEM further analyzed the microstructures of the electroplated Cu foils. A time-of-flight secondary ion mass spectrometer (TOF–SIMS V, ION-TOF, Germany) was used to analyze the intensities of the impurities (carbon, sulfur, Cl, and oxygen) in the electroplated Cu foils.

## Results and discussion

**Figure 3:**
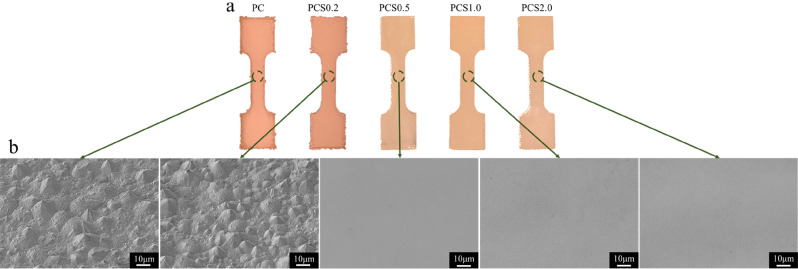
Top-view morphologies of various Cu electroplated layers captured by (**a**) an optical microscope and (**b**) a SEM.

Figure 3a shows the top-view optical images of the electroplated Cu foils peeled from the glass substrate after electroplating with SPS concentrations of 0, 0.2, 0.5, 1.0, and 2.0 ppm. The specimens were labelled as PC, PCS0.2, PCS0.5, PCS1.0, and PCS2.0, respectively. Although the top-view morphology of PCS0.2 is very similar to that of PC, the images show that the surface brightness of the Cu foil was significantly enhanced by increasing the SPS concentration. This is because the increase in the concentration of SPS gradually replaced the PEG molecules (suppressor) attached to the electroplated surface, accelerating the reduction of Cu ions^13,14^. When the concentration of SPS was low (0.2 ppm), the effect of the accelerator on the electroplating was very limited; therefore, the morphologies of PC and PCS0.2 resembled each other. When the concentration of SPS was increased to 0.5 ppm, the SPS molecules began to affect the Cu reduction. An increase in Cu reduction provided a uniform electroplating rate on the electroplated surface at the cathode to lower the roughness of the electroplated Cu surface. The SPS was also referred to as a brightener, and the Cu foils of PCS0.5–2.0 were brighter than those of PC and PCS0.2. The effect of SPS on the roughness of the electroplated Cu foil is illustrated by the SEM images in Fig. 3b. The top-view morphology of PC was very rough and had large cone structures, and the size of the cones was significantly reduced by 0.2 ppm SPS. Furthermore, the cones mostly disappeared when the concentration of SPS was ≥ 0.5 ppm, with the electroplated surface being very smooth. Excellent surficial uniformities of PCS0.5–2.0 were be observed in the higher-magnification SEM images (× 10,000), as shown in Fig. S1. Although the rough surface could be improved through an electropolishing process following electroplating^21^, the different microstructures with varying SPS concentrations possibly impacted the mechanical properties of the electroplated Cu foil.

**Figure 4:**
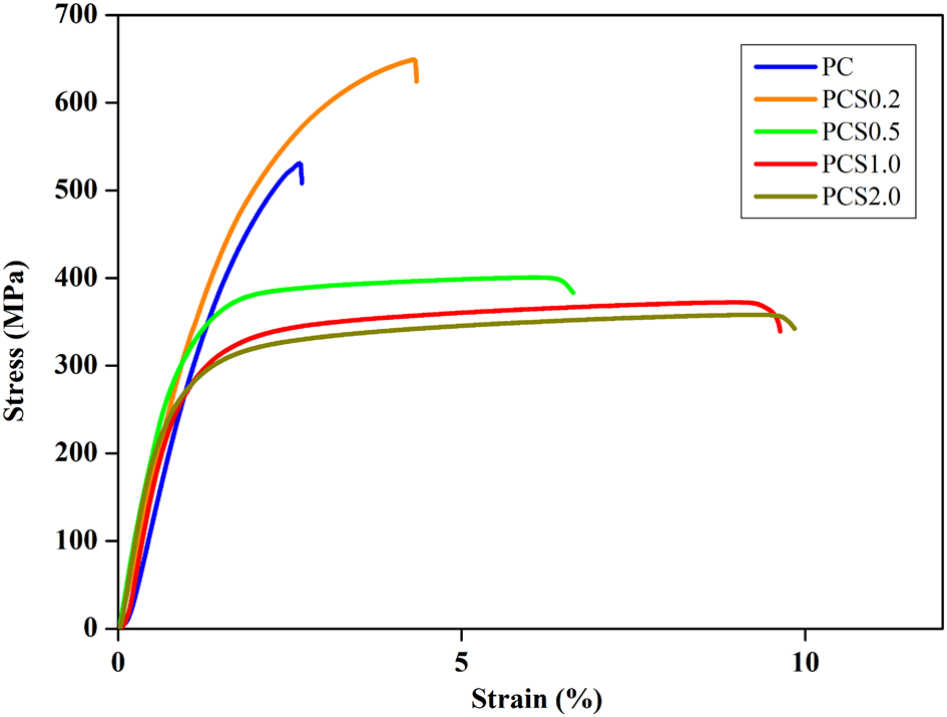
Stress–strain curves of the Cu electroplated layers obtained through tensile tests and labelled as (**a**) PC, (**b**) PCS0.2, (**c**) PCS0.5, (**d**) PCS1.0, and (**e**) PCS2.0.

**Figure 5:**
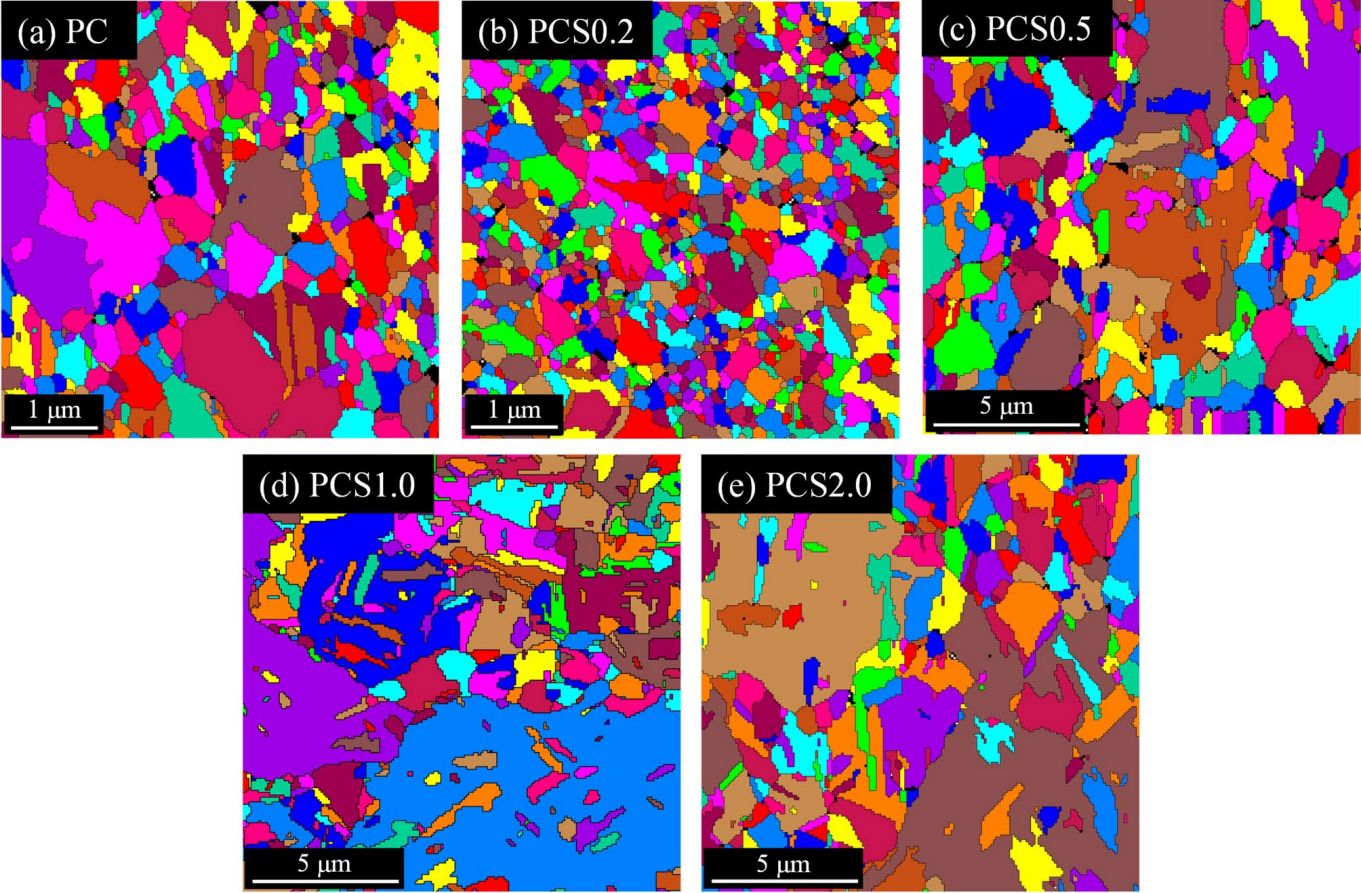
EBSD grain mappings of the Cu electroplated layers labelled as (**a**) PC, (**b**) PCS0.2, (**c**) PCS0.5, (**d**) PCS1.0, and (**e**) PCS2.0. The mappings are created by OIM Analysis v8 (https://www.edax.com/products/ebsd/oim-analysis).

**Figure 6:**
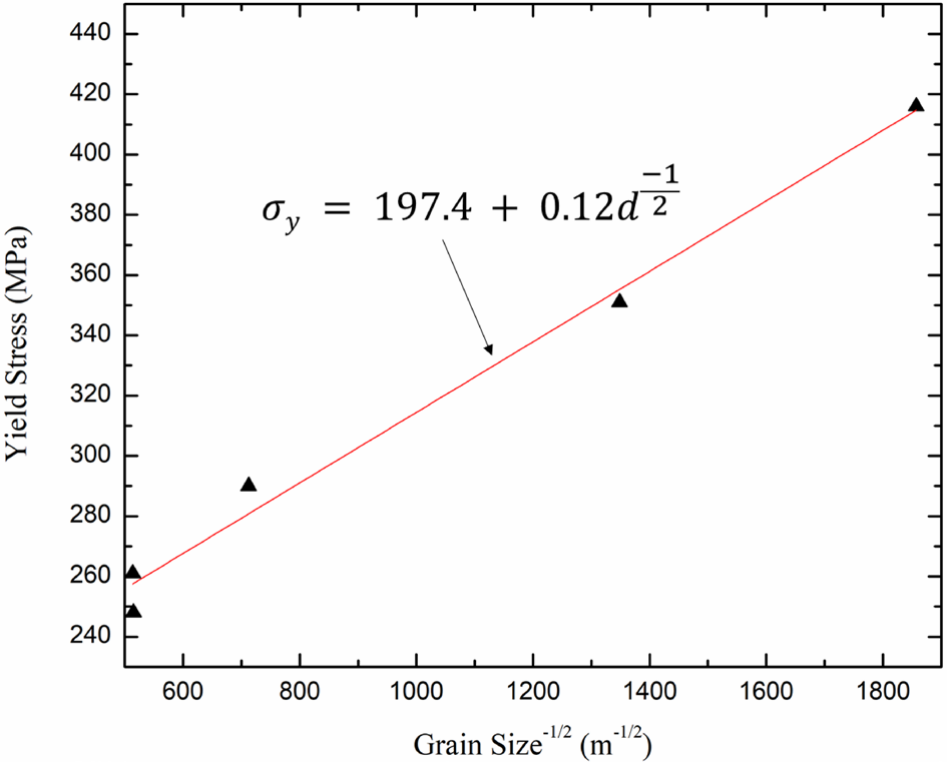
Yield stress-grain size relationship of the electroplated Cu foils.

Tensile tests were performed on the electroplated Cu foils to measure their yield stresses and elongations. The stress–strain curves illustrated in Fig. 4 reveal the different mechanical performances of the foils electroplated with different solutions. Foils with rough surfaces (PC and PCS0.2) exhibited higher yield strengths than those with smooth surfaces (PCS0.5–2.0), and PCS0.2 exhibited the highest tensile strength. Conversely, the elongation of the latter was better than that of the former. Table 1 summarizes the average yield stress and elongation of the five tensile specimens for each electroplating condition. The average yield stress of PCS0.2 was the highest (416 MPa), and the average elongation of PCS2.0 was the highest while that of PCS1.0 was very close to it. The PCS0.5 exhibits intermediate yield strength and elongation among the foils. The trend of these results primarily corresponds to that of the stress–strain curves shown in Fig. 4. In a polycrystalline metal, the strengthening mechanism is mainly attributed to the crystalline size and concentration of impurities^24^. The SEM images shown in Fig. 3b indicate a decrease in the size of the cones when 0.2 ppm SPS was added to the electroplating solution of PC. If the cones can be regarded as multiple grains, the grain size difference in the Cu foils can be changed by the addition of SPS. Thus, grain size reduction is one of the main reasons to reinforce the foils^25^. This is because metallic deformation requires the movement of the dislocations in the metal, and the grain boundary is an obstacle that blocks the dislocation movement. If there were more grain boundaries, that is, a smaller grain size to significantly stop the dislocation slips, the strength of the deformed metal was enhanced, and this phenomenon was referred to as the Hall–Petch effect^26^. The Hall–Petch equation can be expressed as follows:

1 $${\sigma }_{y}={\sigma }_{y,0}+k{d}^{\frac{-1}{2}},$$

where ${\sigma }_{y}$ denotes the yield strength that varies with the grain size, ${\sigma }_{y,0}$ is the original yield stress, *k* is a constant, and *d* is the grain size^27^. Moreover, EBSD can precisely analyze the average grain size of electroplated Cu foils^28^. Figure 5 shows the EBSD mapping of the grains in the electroplated Cu foil. The grains in PC were small but slightly larger than those in PCS0.2, and the grain size increased with increasing SPS concentration. Table 2 summarizes the grain sizes of the Cu foils. The electroplated Cu of PCS0.2 exhibits the smallest grain size (0.29 μm) among all the foils and the highest strength. When the grain size was on the nanoscale, the dislocation slips quickly encountered grain boundaries, inducing the Cu foils of PC and PCS0.2 to reinforce and deform. Thus, they exhibited high strengths and low elongations^29^. Conversely, the grain sizes of PCS1.0 and PCS2.0, which were ten times larger than those of PCS0.2, exhibited higher elongations. Additionally, PCS0.5, with an intermediate grain size, exhibited medium strength and elongation. Figure 6 illustrates the yield stress data with the inverse square root of the grain size. The linear fitting for the data points was ${\sigma }_{y}=197.4+0.12{d}^{\frac{-1}{2}}$, whose tendency certainly met the Hall–Petch effect. The constant *k* of the electroplated Cu in this study was 0.12 MPa m^1/2^, which is close to that obtained in a previous study (0.14 MPa m^1/2^)^30^. Boundary strengthening was demonstrated by the mechanical properties of the electroplated Cu foils. In contrast, the X-ray diffraction patterns in Fig. S2 exhibit that the grain orientations were distributed randomly in those Cu foils. The effect of Cu grain orientation on the mechanical properties of the Cu foils can be ignored, and the Hall–Petch equation was very suitable for evaluating the strengths of the foils.

**Table 1:** Yield stresses and elongations of various Cu electroplated samples.

Specimens	PC	PCS0.2	PCS0.5	PCS1.0	PCS2.0
Yield stress (MPa)	351 ± 8	416 ± 8	290 ± 12	261 ± 5	248 ± 2
Elongation (%)	2.9 ± 0.2	4.3 ± 0.3	7.2 ± 0.9	9.0 ± 0.7	9.4 ± 0.4

**Table 2:** Average grain sizes of various Cu electroplated samples determined by the EBSD grain mappings in Fig. 5.

Specimens	PC	PCS0.2	PCS0.5	PCS1.0	PCS2.0
Grain size (μm)	0.55	0.29	1.97	3.79	3.77

**Figure 7:**
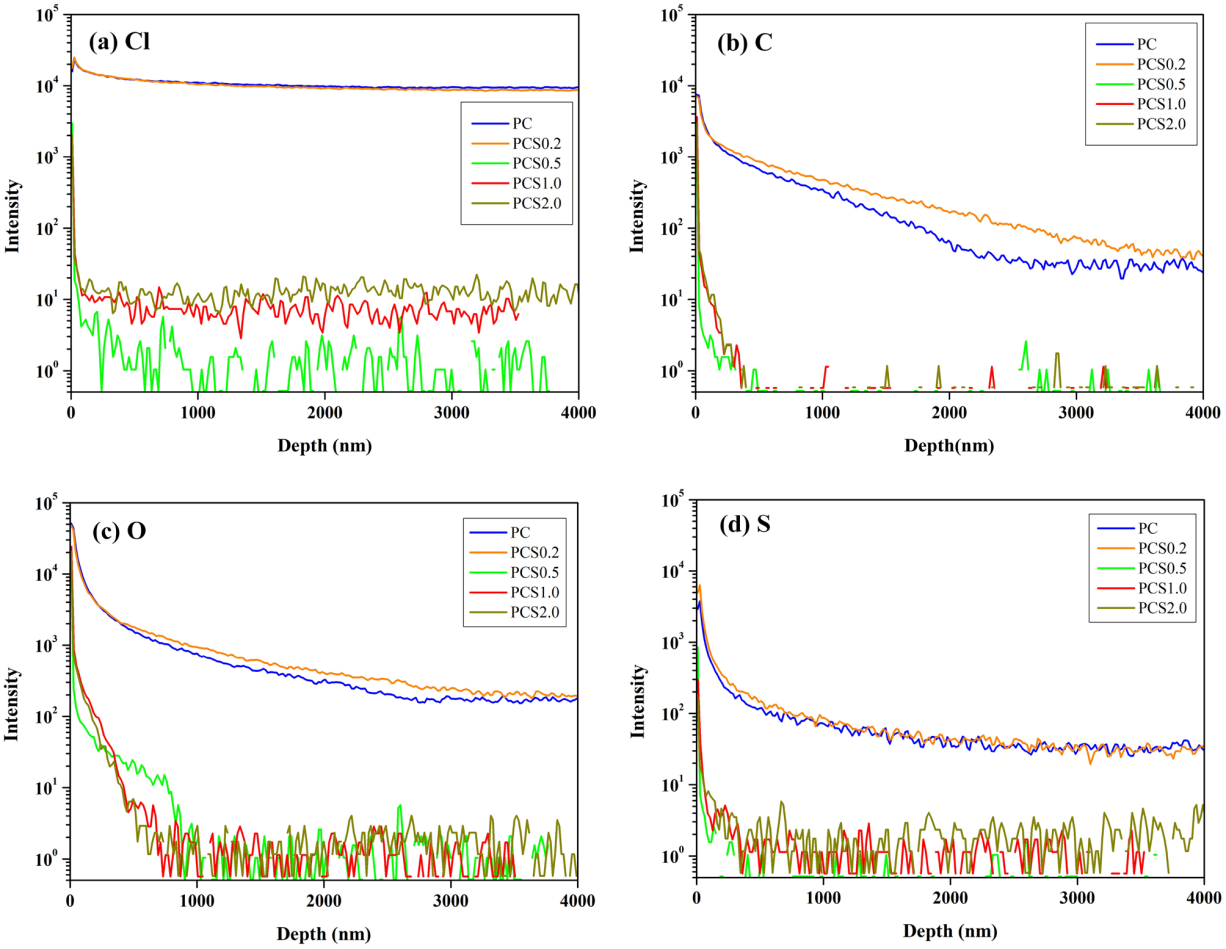
SIMS intensity of (**a**) Cl, (**b**) C, (**c**) O, and (**d**) S as a function of detection depth in the Cu electroplated layers labelled as PC–PCS2.0.

**Figure 8:**
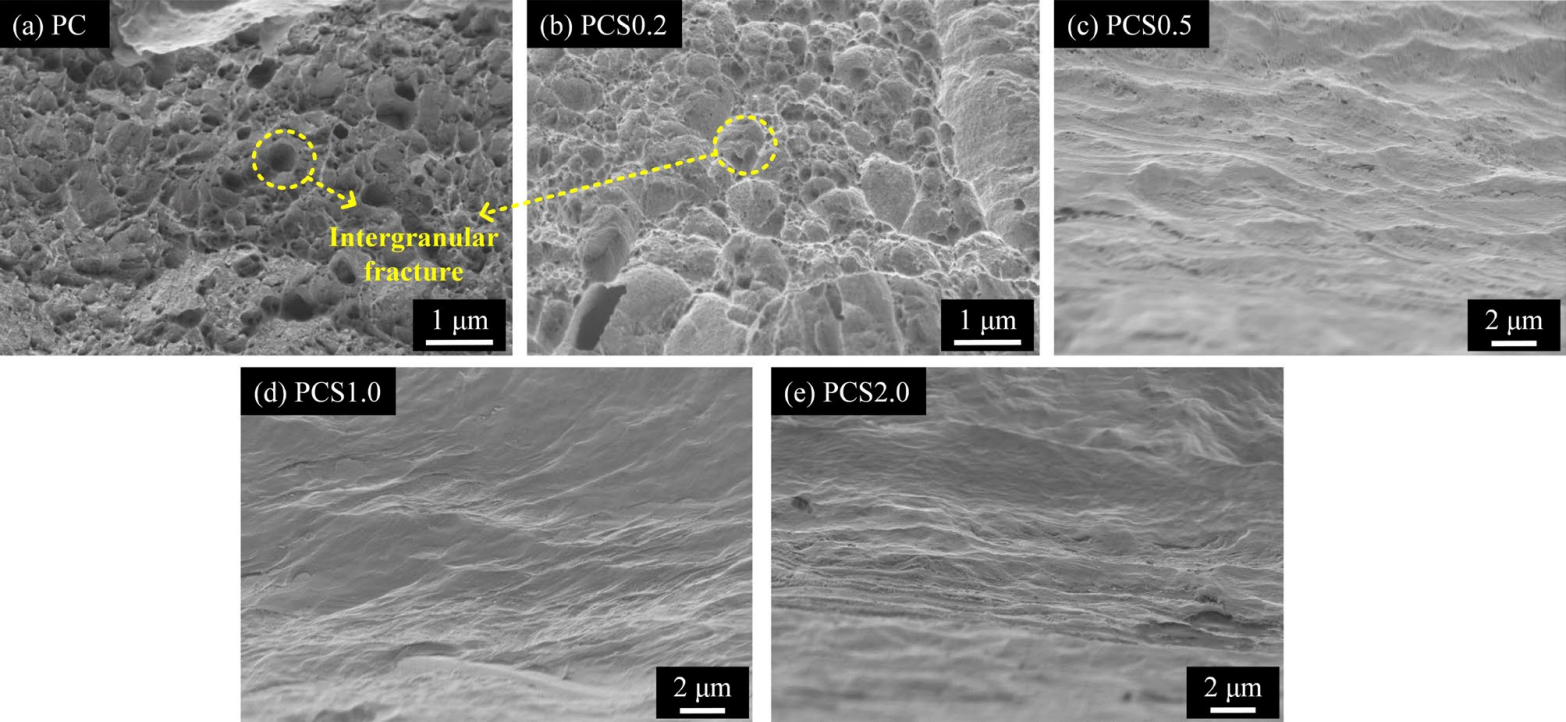
Fracture surfaces of Cu electroplated layers labelled as (**a**) PC, (**b**) PCS0.2, (**c**) PCS0.5, (**d**) PCS1.0, and (**e**) PCS2.0 after tensile tests.

The difference in grain size can be attributed to the impurities in the electroplated Cu foils. Impurities originating from the electrolytes and additives of the plating solutions were inevitably co-deposited with the reduced Cu atoms and were likely to exist at the crystalline boundaries in the as-electroplated Cu^5,9^. The impurities at the boundaries suppressed the growth of the electroplated Cu crystals; that is, the impurities pinned the movement of the boundaries during crystal growth owing to the drag effect. Moreover, the process occurred at room temperature and did not provide sufficient kinetic energy to migrate the grain boundaries blocked by the impurities. Therefore, if there are numerous impurities in the electroplated Cu, the grain size is typically small^31^. This inference is supported by SIMS analysis of chloride, carbon, sulfur, and oxygen in the electroplated Cu foils of PC, PCS0.2, PCS0.5, PCS1.0, and PCS2.0, as shown in Fig. 7. The PC and PCS0.2 samples with smaller grain sizes incorporated more impurities than PCS0.5, PCS1.0, and PCS 2.0, which had larger grain sizes. In particular, PCS0.2, which had the smallest grain size, contained the highest intensities of C and O. The results demonstrated the effect of impurities on the Cu grain size and mechanical properties of the electroplated Cu. Figure 8 shows the fracture surfaces of PC, PCS0.2, PCS0.5, PCS1.0, and PCS2.0 after the tensile test. Several dimple structures were observed on the fracture surfaces of PC and PCS0.2 Cu. The dimple structures surrounded by grains were morphological after an intergranular fracture. When impurities accumulated at the grain boundaries in the Cu foils, the grain boundaries became significantly weak points for stress concentration. Consequently, we observed intergranular fractures in Cu foils with a high concentration of impurities. In contrast, PCS0.5–2.0 Cu with large grain sizes contained a significantly low concentration of impurities, and the fracture surfaces with the extension of grain boundaries owing to tensile stress exhibited a transgranular fracture mode^32^. The fracture modes correspond to the SIMS analysis.

## Conclusions

In this study, we examined the mechanical properties of electroplated Cu foils with SPS concentrations ranging from 0 to 2.0 ppm. The top-view optical images illustrated that an increase in SPS improved the brightness owing to the improvement in the roughness of the electroplated surface. In the tensile tests, SPS0.2 Cu exhibited the highest yield strength, whereas SPS1.0 and 2.0 exhibited significant elongations. According to the EBSD analysis, the grain size in the latter was ~ 10 times larger than that in the former. The Hall–Petch effect on the mechanical properties of the electroplated Cu was significant and it obeyed the linear fitting of ${\sigma }_{y}=197.4+0.12{d}^{\frac{-1}{2}}$. The small grain size of electroplated Cu was attributed to the high concentration of impurities identified through SIMS. When more impurities were present at the grain boundaries, the Cu foils grain size was small. Impurities at the grain boundaries affected not only the grain size but also the fracture mode in the tensile foils. Cu foils with low and high concentrations of impurities were broken by transgranular and intergranular fractures, respectively. The obtained results demonstrated that the SPS concentration controlled the microstructures of the electroplated Cu, resulting in a significant Hall–Petch effect on the mechanical properties.

## Supplementary Information

Supplementary Figures.

## Data Availability

The datasets used and analyzed in this study are available from the corresponding author upon reasonable request.

## References

[1] Murarka SP, Hymes SW (1995). Copper metallization for ULSL and beyond. Crit. Rev. Solid State Mater. Sci..

[2] Chen HP (2016). Optimization of the nanotwin-induced zigzag surface of copper by electromigration. Nanoscale.

[3] Andricacos PC, Uzoh C, Dukovic JO, Horkans J, Deligianni H (1998). Damascene copper electroplating for chip interconnections. IBM J. Res. Dev..

[4] Shen Y-A (2022). Graphene as a diffusion barrier at the interface of Liquid-State low-melting Sn–58Bi alloy and copper foil. Appl. Surf. Sci..

[5] Chan P-F (2020). Effect of copper grain size on the interfacial microstructure of a Sn/Cu joint. ACS Appl. Electron. Mater..

[6] Zheng Z (2019). Study of grain size effect of Cu metallization on interfacial microstructures of solder joints. Microelectron. Reliab..

[7] Wang Y, Huang Y-T, Liu YX, Feng S-P, Huang MX (2022). Thermal instability of nanocrystalline Cu enables Cu-Cu direct bonding in interconnects at low temperature. Scr. Mater..

[8] Hsiao H-Y (2012). Unidirectional growth of microbumps on (111)-oriented and nanotwinned copper. Science.

[9] Chiang P-C, Shen Y-A, Feng S-P, Chen C-M (2021). Electrodeposition of twinned Cu with strong texture effect on voiding propensity in electroplated Cu solder joints. J. Electrochem. Soc..

[10] Nagy Z, Blaudeau JP, Hung NC, Curtiss LA, Zurawski DJ (1995). Chloride ion catalysis of the copper deposition reaction. J. Electrochem. Soc..

[11] Hebert KR (2005). Role of chloride ions in suppression of copper electrodeposition by polyethylene glycol. J. Electrochem. Soc..

[12] Dow W-P, Yen M-Y, Lin W-B, Ho S-W (2005). Influence of molecular weight of polyethylene glycol on microvia filling by copper electroplating. J. Electrochem. Soc..

[13] Kondo K, Matsumoto T, Watanabe K (2004). Role of Additives for copper damascene electrodeposition: Experimental study on inhibition and acceleration effects. J. Electrochem. Soc..

[14] Moffat TP, Wheeler D, Josell D (2004). Electrodeposition of copper in the SPS-PEG-Cl additive system: I Kinetic measurements: Influence of SPS. J. Electrochem. Soc..

[15] Ramm P, Klumpp A, Weber J, Taklo MMV (2009). 3D system-on-chip technologies for More than Moore systems. Microsyst. Technol..

[16] Tu KN (2011). Reliability challenges in 3D IC packaging technology. Microelectron. Reliab..

[17] Chung, S., Chen, Y. T. & Chen, Z. C. Electroplated nanotwin copper for fine line RDL. *China Semicond. Technol. Int. Conf. 2018, CSTIC 2018* 1–3. 10.1109/CSTIC.2018.8369296 (2018).

[18] Lau JH (2018). Fan-out wafer-level packaging. Fan-Out Wafer-Level Packag.

[19] Yu CK (2017). A unique failure mechanism induced by chip to board interaction on fan-out wafer level package. IEEE Int. Reliab. Phys. Symp. Proc..

[20] Liu WT (2021). A fracture mechanics evaluation of the Cu-polyimide interface in fan-out redistribution Interconnect. Proc. Electron. Compon. Technol. Conf..

[21] Li YJ, Tu KN, Chen C (2020). Tensile properties of <111>-oriented nanotwinned Cu with different columnar grain structures. Materials.

[22] Li YJ, Hsu WY, Lin B, Chang C, Chen C (2019). High-Toughness (111) nano-Twinned copper lines for fan-out wafer-level packaging. Int. Conf. Electron. Packag..

[23] Lin YM, Lee CY, Chen YL, Pan CP, Ho CE (2022). Significantly improving the mechanical/electrical characteristics of blind-hole Cu filling through crystal coherent modification. Surf. Coat. Technol..

[24] Abbaschian R, Abbaschian L, Reed-Hill ER (2010). Physical Metallurgy Principles.

[25] Lu L, Shen Y, Chen X, Qian L, Lu K (2004). Ultrahigh strength and high electrical conductivity in copper. Science.

[26] Liu M (2008). Study of the interaction between the indentation size effect and Hall-Petch effect with spherical indenters on annealed polycrystalline copper. J. Phys. D. Appl. Phys..

[27] Mouritz AP, Mouritz AP (2012). Strengthening of metal alloys. Introduction to Aerospace Materials.

[28] Chen KJ, Wu JA, Chen C (2020). Effect of reverse currents during electroplating on the ⟨111⟩-oriented and nanotwinned columnar grain growth of copper films. Cryst. Growth Des..

[29] Lu K, Lu L, Suresh S (2009). Strengthening materials by engineering coherent internal boundaries at the nanoscale. Science.

[30] Hansen N (2004). Hall–Petch relation and boundary strengthening. Scr. Mater..

[31] Chiang P-C, Shen Y-A, Chen C-M (2021). Effects of impurities on void formation at the interface between Sn-3.0Ag-0.5Cu and Cu electroplated films. J. Mater. Sci. Mater. Electron..

[32] Casari D, Merlin M, Garagnani GL (2013). A comparative study on the effects of three commercial Ti-B-based grain refiners on the impact properties of A356 cast aluminium alloy. J. Mater. Sci..

