# Supplementary material for: Significant Hall–Petch effect in micro-nanocrystalline electroplated copper controlled by SPS concentration
Source: Sci Rep. 2023 Jan 9;13:428. doi: 10.1038/s41598-023-27669-2 (PMC9829657; doi:10.1038/s41598-023-27669-2)
Supplement: Supplementary file 1 — Supplementary Figures. [file 41598_2023_27669_MOESM1_ESM.docx]

Significant Hall-Petch Effect in Micro-Nanocrystalline Electroplated Copper Controlled by SPS Concentration

Yu-Jyun Kao ^1^, Yu-Ju Li^1^, Yu-An Shen ^2,^*, Chih-Ming Chen ^1,3,^*

^1^ Department of Chemical Engineering, National Chung Hsing University, 145 Xingda Rd., South Dist., Taichung 402, Taiwan

^2^ Department of Materials Science and Engineering, Feng Chia University, No. 100, Wenhwa Rd., Seatwen, Taichung 407, Taiwan

^3^ Innovation and Development Center of Sustainable Agriculture (IDCSA), National Chung Hsing University, 145 Xingda Rd., South Dist., Taichung 402, Taiwan

*Corresponding authors

E-mail address: chencm@nchu.edu.tw (C.M. Chen)

Tel.: +886-4-22840510 ext. 511

E-mail address: [yashen@fcu.edu.tw](mailto:yashen@fcu.edu.tw) (Y.A. Shen)

Tel.: +886-4-24517250 ext. 5309


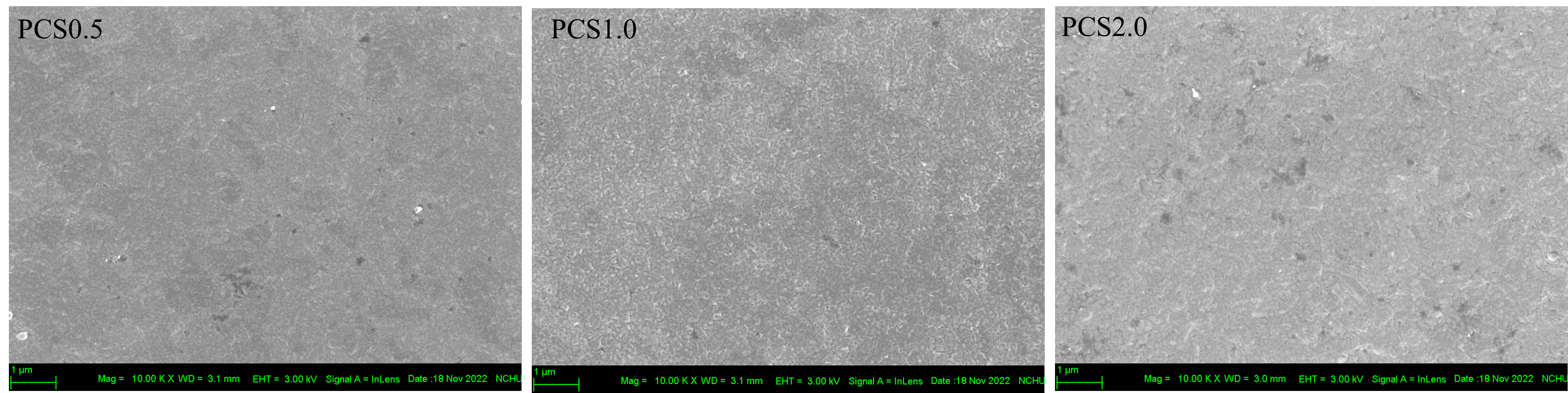


Figure S1: The SEM images in higher magnification (10,000x), showing the good uniformities of surface roughness in PCS0.5-PCS2.0

Figure S2: The XRD patterns in the Cu foils of PC-PCS2.0. The scanning rate of the XRD was 2$^{\circ}$/min.
